# Supplementary material for: Proposal of a guide for the interpretation, simplification of the regulatory process and good tax compliance, case of digital taxpayers, influencers
Source: PLoS One. 2023 Jun 16;18(6):e0286617. doi: 10.1371/journal.pone.0286617 (PMC10275450; doi:10.1371/journal.pone.0286617)
Supplement: S2 File — (DOCX) [file pone.0286617.s002.docx]

**INTERVIEW GUIDE INFLUENCER**

Presentation of the Interviewer and Influencer (Introductory Phase)

*Main question 1:* What motivates a person to become an influencer.

Who or how would you describe yourself?

How has your trajectory been in social networks according to your activity (tell us your story)?

1. How did you get started in the world of social media?

2. What is your profession? What do you do now?

3. What prompted or motivated you to become an INFUENCER or content generator?

4. Did you think that the success you have in your videos or publications would come so fast?

5. Do you consider the role of the INFLUENCER or content generator in social networks to be important nowadays?

6. How would you describe the work that influencers do?

Main question 2.

Advertising phase for other companies or revenue generators

How do you monetize on social networks?

How do you carry out the INFLUENCER activity on social networks?

7. Which social network do you use the most to advertise your videos and make your opinion of brands known? and why?

8. How often do you make videos and/or posts on your social networks?

9. From which social media activities do you earn your income?

10. How was your experience promoting something on your networks?

11. Have you collaborated with any fashion brand or other specific brand?

12. Do you think that the role of INFLUENCER in a marketing strategy is favorable in increasing sales?

13. Do you consider that the videos you make influence the purchase decision of your 14. followers (in this case, the videos you make)?

14. your followers (in this case the MSEs)?

15. Has any Sponsor (Sponsor) asked you for any document for your advertising service?

16. Have they ever asked you for an advertisement where you speak well of the product or service?

17. Do web platforms send you money as payment for your content?

Main question 3.

Entrepreneurial stage, business and tax knowledge

INFLUENCER's entrepreneurial capacity and to what extent does INFLUENCER know about tax issues?

What is your purpose in the future with the increase of your followers, what is your goal?

18. Would you dedicate any professional work (entrepreneurship) to the creation of your content?

19. Do you think that the figure of the INFLUENCER could reach the level of a profession?

20. Are you a designer and do you have your own brand? If so, what marketing actions do you think MSEs should carry out for their growth? And what would you plan to implement in your company or future venture to be more successful?

21. Do you create your content alone or do you need support to create your content?

22. What purpose do you have in the future with the increase of your followers, what is your goal.

23. In your opinion as an INFLUENCER or content generator, do you think it is fair or reasonable that the state levies taxes or imposes a tax rate (%) for your activities or income (profits) that you generate.

24. Do you have any tax knowledge?

25. Would you like to have a clear guide for incorporating the tax system? If so, why?

26. If you had access to a guide on the payment of taxes for your advertising services, would you pay taxes or if you are already declaring your taxes, how do you do it?
