# Supplementary material for: Proposal of a guide for the interpretation, simplification of the regulatory process and good tax compliance, case of digital taxpayers, influencers
Source: PLoS One. 2023 Jun 16;18(6):e0286617. doi: 10.1371/journal.pone.0286617 (PMC10275450; doi:10.1371/journal.pone.0286617)
Supplement: S3 File — (DOCX) [file pone.0286617.s003.docx]

**INTERVIEW GUIDE TAX EXPERT**

1. **Introduction:**

We are in the process of developing qualitative research on the tax obligations of "Influencers"; for this reason we would like to know your opinion about different tax aspects in this group of taxpayers.

We thank you in advance for your kind attention. Thank you.

1. **Questions:**

- Do you believe that our current regulations tax the income produced by the various activities of the "Influencers" or do they present legal loopholes on the subject; if so, what is the current tax treatment for these taxpayers, both national and foreign, to comply with the payment of their taxes in our country?
- In your opinion, do you consider that the current regulatory framework is ideal for this type of taxpayers "Influencers" or what would be the modifications that you would recommend to the legislator for its optimization?
- Do you agree with the current auditing procedures established by the tax administration or what are the procedures and strategies that you would recommend to avoid evasion in this sector, and on the contrary, to generate higher levels of collection?
