# Supplementary material for: Proposal of a guide for the interpretation, simplification of the regulatory process and good tax compliance, case of digital taxpayers, influencers
Source: PLoS One. 2023 Jun 16;18(6):e0286617. doi: 10.1371/journal.pone.0286617 (PMC10275450; doi:10.1371/journal.pone.0286617)
Supplement: S1 Appendix — (PDF) [file pone.0286617.s004.pdf]

S1 Appendix

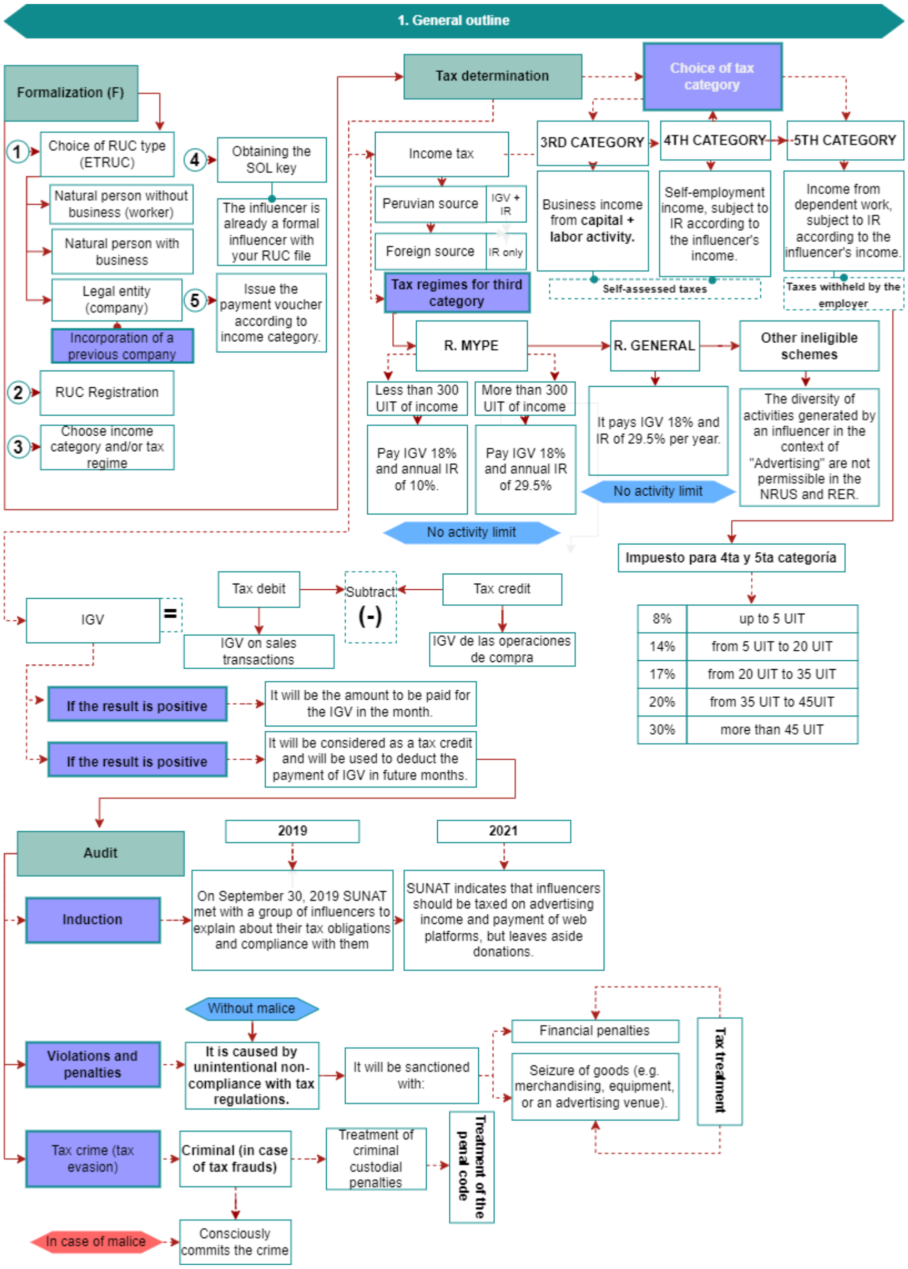

Fig 3. Guide for the interpretation, simplification of the regulatory process and good tax compliance
