# Supplementary material for: Proposal of a guide for the interpretation, simplification of the regulatory process and good tax compliance, case of digital taxpayers, influencers
Source: PLoS One. 2023 Jun 16;18(6):e0286617. doi: 10.1371/journal.pone.0286617 (PMC10275450; doi:10.1371/journal.pone.0286617)
Supplement: S2 Appendix — (PDF) [file pone.0286617.s005.pdf]

S2 Appendix

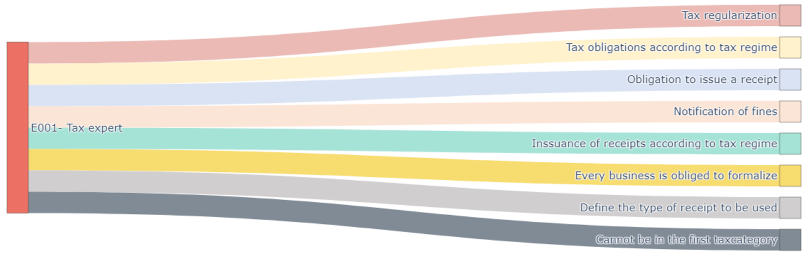

Fig 4. Sankey diagram tax expert 001

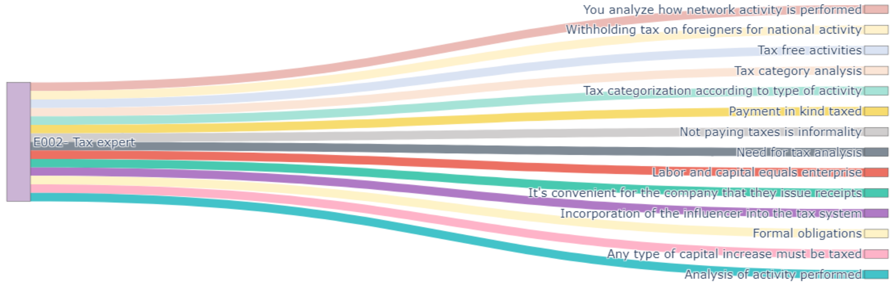

Fig 5. Sankey diagram tax expert 002

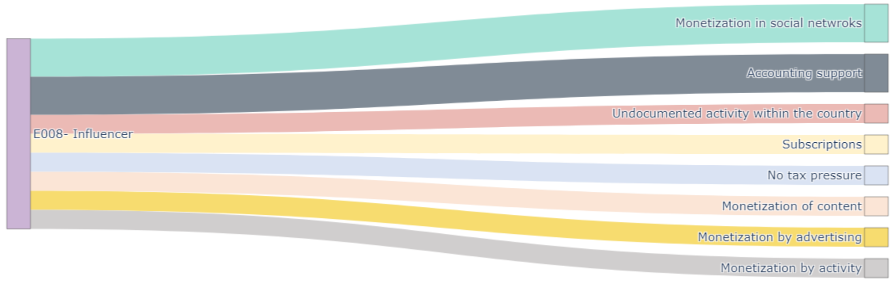

Fig 6. Sankey diagram influencer 008

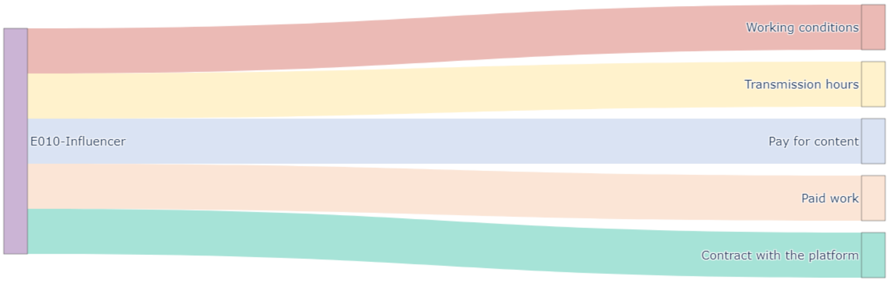

Fig 7. Sankey diagram influencer 010

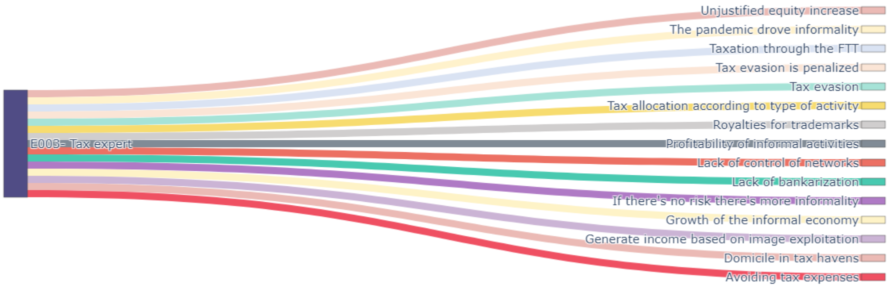

Fig 8. Sankey diagram tax expert 003
