# Supplementary material for: Proposal of a guide for the interpretation, simplification of the regulatory process and good tax compliance, case of digital taxpayers, influencers
Source: PLoS One. 2023 Jun 16;18(6):e0286617. doi: 10.1371/journal.pone.0286617 (PMC10275450; doi:10.1371/journal.pone.0286617)
Supplement: Attachment — Submitted filename: Rebutal letter.pdf [file pone.0286617.s006.pdf]

Subject: Submission of revised paper (PONE-D-22-01029)

April 5, 2023

Jaime A. Yáñez, Ph.D.
Plos One

Dear Editor

Thank you for allowing us to submit a revised draft of our manuscript titled "Proposal of a guide for the interpretation, simplification of the regulatory process and good tax compliance, case of digital taxpayers, influencers" We appreciate the time and effort that you and the reviewers have dedicated to providing your valuable feedback on our manuscript.

We are grateful to the reviewers for their insightful comments on our paper. We have been able to incorporate changes to reflect most of the suggestions provided by the reviewers. We have highlighted the changes within the manuscript. We are sure that our paper's quality and legibility have significantly improved thanks to the reviewers' comments.

Reviewer 1

1.1 — Influencers are not opinion leaders. Please, review in deep the details to explain better the concept.

Reply: the change was made and the following was written in the first paragraph of the abstract and introduction: "Influencers generate opinions in individuals through multiple virtual platforms", using the author DeMarzo PM, Vayanos D, Zwiebel J. Persuasion Bias, Social Influence, and Unidimensional Opinions*. The Quarterly Journal of Economics. 2003;118(3):909–968. doi:10.1162/00335530360698469.

1.2 — I think ithe aim must be an analysis. Make a guide NOT is a research outcome.

Reply: the change was made from line 33 to line 40 of the introduction, considering pertinent the reviewer's observation, since first an analysis of the tax regulatory framework was made and then the analysis of the experts' and influencers' answers.

1.3 — In methdology is mentioned "have a significant number of followers in social 46 networks". What means "significant number"? 1000 followers? 10 000? 100 000? one million?

Reply: The phrase "have a significant number of followers" was specified in the sample inclusion section in materials and methods; in line 51 where an average number of followers among the 10 influencers of 500,000 is specified.

1.4 — I suggest process the data by ATLAS.TI or other qualitative software.

Reply: The data analysis was performed by entering the documents of the answers for each respondent and their related codes using the AtlasTi software, obtaining in the analysis section of the software the occurrences of document, code and illustrating them in sankey diagrams of the software. These results were added in the results report, in annex 02 and their explanation in lines 95 to 104.

1.5 — he topic is interesintng but need to improve the method of the study

Reply: Once again, thank you very much for your pertinent comments in order to obtain a quality paper. The wording of the Scribber steps was improved, explaining in greater detail steps 01 (transcription of the videos), 02 (process of formulating meanings of the key phrases through qualitative coding), 03 (the themes defined through the group of meanings of the key phrases and that were identified as findings), 04 (explanation of the analysis carried out to obtain the tax guide as a product, accompanied with the data analysis of Atlas ti).

Reviewer 2

2.1 — The title ("Proposal of a guide for the interpretation, simplification of the regulatory process and good tax compliance, case of digital taxpayers, influencers") and the summary present a correct structure, with an adequate identification of what is subsequently addressed in the text. It is an article that contributes to society and helps to promote knowledge about the importance of digital taxpayers and their taxation, from the point of view of influencers. The objectives and hypotheses are well stated and appropriate, with methodological rigour and well-designed and thought-out research instruments. Therefore, the results of the review are evident and relevant. The results are clearly presented, leading to a discussion section and conclusions that correlate with the objectives and methodology employed, as well as with the results obtained, all in a transparent and convincing manner. It is a good article to which I would perhaps add some more global perspective and updated references. In this regard, I recommend citing the following references

Reply: Authors were added to the introduction section, very important to improve the context of the research on lines 2, 9 and 10
- DeMarzo, P., Vayanos, D., Zwiebel, J. (2003). Persuasion bias, social influence, and unidimensional opinions. The Quarterly Journal of Economics, 118(3), 909- 968.
- Di Gioacchino, D., Fichera, D. (2022). Tax evasion and social reputation: The role of influencers in a social network. Metroeconomica, 73(4), 1048-1069.

We hope the revised version fulfills the expectations of the editorial team and look forward to hearing from you in due course.

Sincerely,

Dr. Jorge Sánchez-Garces
Professor
Faculty of Engineering and Management
NATIONAL TECHNOLOGICAL UNIVERSITY OF SOUTH LIMA, LIMA, PERÚ
E-mail: jsanchezg@untels.edu.pe

On behalf of all authors.
